# Seroepidemiology of pertussis in the east of China: Estimates of incidence of infection in adolescents and adults pre- and post-COVID-19

**DOI:** 10.3389/fpubh.2022.1054617

**Published:** 2022-12-01

**Authors:** Qiang Chen, Wen Wang, Xiuyun Shi, Yan Xu, Yanhong Zhu, Yun Wu, Zhiguo Wang, Hui Sun, Xiang Sun

**Affiliations:** ^1^Expanded Program on Immunization, Jiangsu Provincial Center for Disease Control and Prevention, Nanjing, China; ^2^Department of Rheumatology and Immunology, The Affiliated Suqian First People's Hospital of Nanjing Medical University, Suqian, China; ^3^Expanded Program on Immunization, Siyang Center for Disease Control and Prevention, Suqian, China; ^4^School of Health Policy and Management, Nanjing Medical University, Nanjing, China; ^5^Medical Department, Affiliated Hospital of Nanjing University of Traditional Chinese Medicine, Nanjing, China

**Keywords:** pertussis, antibodies, vaccination, seroprevalence, adolescents and adults

## Abstract

**Introduction:**

The dramatic decrease in the number of reported cases of pertussis during COVID-19 pandemic has been underestimated. The objective was to compare the estimated incidence rate of pertussis in populations pre- and post-COVID-19 pandemic by analyzing the anti-pertussis toxin (anti-PT) IgG and anti-filamentous hemagglutininant (anti-FHA) IgG antibodies in healthy Chinese population from 2018 to 2021.

**Methods:**

All serum samples (*N* = 1,000) were collected from healthy population (aged ≥ 15 years) who attended an annual monitoring project of antibody levels in Jiangsu province in 2018–2021 were measured by ELISA.

**Results:**

The positive rates of anti-PT IgG and anti-FHA IgG antibodies were 11.4% (114/1,000) and 20.2% (202/1,000) (≥40 IU/ml), the GMC were 17.25 (95% CI: 15.49–19.03) IU/mL and 24.94 (95% CI: 22.73–27.16) IU/mL in the study population, respectively. The percentage of participants with anti-PT IgG antibodies higher than 40 IU/mL was 5.20% (11/212) in 2018, 5.5% (19/348) in 2019, 21.2% (46/217) in 2020 and 17.0% (38/223) in 2021, respectively. The non-detectable rate (<5 IU/mL) of anti-PT IgG antibodies was 16.9, 17.7, 28.1, and 37.3% in 2018, 2019, 2020, and 2021, respectively. We assumed that the infection occurred within 58.6 days, and based on the overall proportion (2.9%) of individuals with anti-PT IgG antibody ≥100 IU/ml, the incidence rate (/100) was estimated by the formula to be 18.08 (95% CI: 12.40–26.11). In addition, the estimated incidence of Post-COVID-19 was higher than that of Pre-COVID-19 (36.33/100 vs. 12.84/100), and the difference was statistically significant (*p* < 0.05).

**Conclusions:**

Our results suggest a high rate of under-reporting of pertussis in Jiangsu Province both pre- and post-COVID-19 pandemic, and there are a large number of adults of childbearing age who are susceptible to pertussis. It seems imperative that vaccination of adolescents and adults should be considered for inclusion in vaccination programs.

## Introduction

Pertussis is a highly infectious disease caused by Bordetella pertussis (1). The disease occurs in all age groups, adolescents, and adults are the main hosts and sources of infection for this pathogen, which can be transmitted by contact to unvaccinated individuals and infants who have not completed their primary vaccination program (2, 3). Since the 1950s, the pertussis vaccine has been administered to large numbers of people globally, throughout the past several decades the prevalence of pertussis has increased in some developed nations, even in those with high vaccination coverage (4, 5). A shift in the epidemiological characteristics of pertussis to adolescent and adult populations has also been found in many developed countries, including China (6–8). However, the number of reported pertussis cases in China decreased significantly during the COVID-19 pandemic, from 30,027 cases in 2019 to 4,475 cases in 2020 (9). Due to the implementation of a series of non-pharmacological interventions (lockdown, wearing masks, maintaining social distance, etc.), which were incredibly successful against the disease caused by respiratory infections (10–13). However, with the COVID-19 pandemic under control, 9,961 cases of pertussis were reported in China in 2021 (9), which was still fewer than the reported number in 2019 (30,027 cases), making it evident that the recurrence of pertussis is still a long-term tendency in China. Many countries recommended booster vaccination for older children (14), but adolescents and adults were still vulnerable, and pertussis in those who had vaccinated manifesting as asymptomatic infection and atypical symptoms like chronic cough, so the actual infection rate may be higher than those reported by clinicians (15–17).

Whole-cell Diphtheria-Tetanus-Pertussis (DTwP) was introduced into the National Immunization Program (NIP) in China in 1978, and to reduce the incidence of adverse reactions and quality control troubles associated with the inability to accurately quantify the effective antigen and other substances, Diphtheria–tetanus–acellular pertussis (DTaP) was registered in 1996 and completely replaced DTwP in 2012 (18). Since 1982, infants have been administered three primary doses of DTwP or DTaP vaccines at the age of 3, 4, and 5 months, with a booster dose given at 18–24 months. After increased surveillance of pertussis-like symptoms in cities such as Tianjin and Shenzhen in China (19, 20), the incidence rate was discovered to be significantly higher than those hospital reporting for the same time period. Several seroepidemiological studies conducted in different Chinese regions that the incidence of pertussis was probably greatly underreported. Jiangsu is a province in eastern China with a population of almost 85 million people in 2021, including roughly 70 million people aged ≥ 15 years. According to a study, in Jiangsu, the number of pertussis cases reported in Jiangsu province had been increasing pre-COVID-19 pandemic, but only 31 cases were reported by 2020 (13), and the true incidence of pertussis (especially in adults) was grossly underestimated.

Seropositivity was not an indicator of prevention against the infection, but can be used as evidence of an immune response to a vaccine or natural infection. Seroepidemiological and antibody prevalence data can help us understand the factors contributing to the resurgence of pertussis. The purpose of this study was to estimate incidence of pertussis in the population before and after the COVID-19 pandemic by analyzing the anti-PT IgG and anti-FHA IgG antibodies in healthy Chinese population from 2018 to 2021, so as to understand the actual infection level of pertussis in the population of Jiangsu, China, as well as to provide suggestions for the optimization of pertussis immunization programs in China.

## Methods

### Subjects and serum samples

All serum samples were collected from healthy individual (aged ≥ 15 years) who participated an annual monitoring project of antibody levels in Jiangsu province in 2018–2021. The annual monitoring project of antibody levels in Jiangsu Province was carried out in Yancheng and Huai'an, and two counties were randomly selected from the two cities to carry out the project, and a total of six counties were selected from 2018 to 2021. Basic demographic characteristics (age, sex, residential area, and date of serum collection) of the subjects were collected. Two milliliter of venous blood was collected, and the serum was separated and stored at −20°C. COVID-19 pandemic broke out in China at the end of 2019, so we defined 2018–2019 as pre-COVID-19 era and 2020–2021 as post-COVID-19 era.

### Laboratory testing

The concentrations of Anti-PT IgG and Anti-FHA IgG antibodies were determined using ELISA kits (Institut Virion/Serion GmbH, Germany). The minimum level of anti-PT IgG detection was 5 IU/ml. Infection with B. pertussis was confirmed if the anti-PT IgG titer was ≥100 IU/mL. ≥40 IU/ml indicated an infection within a few years, and <40 IU/ml was considered as negative.

### Assessment of pertussis infection

de Melker et al. (21) found that it took an average of 58.6 (95% CI: 54.2–63.2) days for the titers of anti-PT-IgG antibody to fall to 100 IU/ml after pertussis infection, and the rate of pertussis infection can be estimated using the formula: 365.25/58.6 × Sero-prevalence (Sero-prevalence: the proportion of subjects with PT-IgG titers ≥100 IU/ml and assuming the infection occurred within 58.6 days), but it represents the rate 1 year before, the numbers for 2021 are used to estimate the incidence for 2020, and so forth. Data for 2022 were unknown and we were unable to estimate the incidence for 2021, so we included only 2020 values in estimating the incidence Post-COVID-19.

### Statistical analysis

Data were analyzed by Microsoft Excel and SPSS V.22.0 (IBM Corp, Armonk, NY). Positive rate was compared using chi-square test, Geometric mean concentration (GMC) was compared using one-way analysis of variance (ANOVA). Non-normally distributed continuous variables of two or more groups were compared using the Mann–Whitney *U*-test or Kruskal–Wallis test. *P* < 0.05 was considered statistically significant.

## Results

### Study population and demographic data

A total of 1,000 serum samples were included in the study, and anti-PT IgG and anti-FHA IgG antibodies were tested in each subject. Two hundred twelve, 348, 217, and 223 participants were collected from 2018 to 2021, respectively; 361 participants were aged 15–19 years, 223 participants were aged 20–29 years, 206 participants were aged 30–39 years and 210 participants were over 40 years. Four hundred eleven males and 589 females were included in the study (Table 1).

**Table 1:** The GMC and positivity rate of anti-PT IgG antibodies in subjects aged ≥ 15 years.

	***N***	**GMC (95% CI) (IU/mL)**	***P*-value**	**Anti-PT IgG (%)**			
				**≥40**	***P-*value**	**≥100**	***P*-value**
Age (years)			0.002		<0.001		0.002
15–19	361	14.15 (11.76–16.54)		27 (7.5)		6 (1.7)	
20–29	223	15.50 (12.48–18.52)		21 (9.4)		3 (1.3)	
30–29	206	17.96 (13.87–22.05)		23 (11.2)		6 (2.9)	
≥40	210	23.52 (18.09–28.94)		43 (20.5)		14 (6.7)	
Sex			0.832		0.976		0.425
Male	411	17.03 (14.13–19.92)		47 (11.4)		14 (3.4)	
Female	589	17.42 (15.17–19.66)		67 (11.4)		15 (2.5)	
Year			0.007		0.016		<0.001
2018	212	13.15 (10.74–15.56)		11 (5.2)		2 (0.9)	
2019	348	15.62 (13.75–17.49)		19 (5.5)		3 (0.9)	
2020	217	21.69 (17.04–26.33)		46 (21.2)		11 (5.1)	
2021	223	19.42 (14.03–24.82)		38 (17.0)		13 (5.8)	
COVID-19			<0.001		<0.001		<0.001
Pre-COVID-19	560	14.68 (13.20–16.16)		30 (5.4)		5 (0.9)	
Post-COVID-19	440	20.54 (16.98–24.09)		84 (19.1)		24 (5.5)	
Total	1,000	17.25 (15.49–19.03)		114 (11.4)		29 (2.9)	

CI, confidence interval.

### Seroprevalence of anti-PT IgG and anti-FHA IgG antibodies

**Figure 1:**
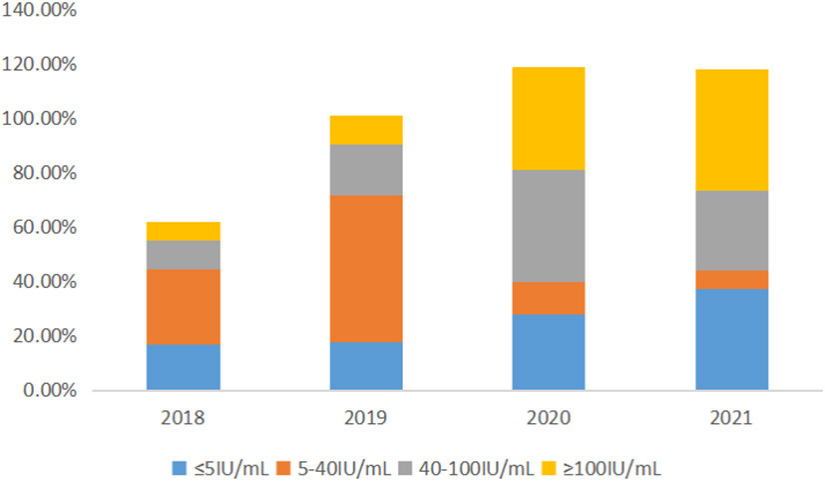
Distribution of serum anti-PT IgG antibody concentrations in Jiangsu, 2018–2021.

The positive rate of anti-PT-IgG antibody was 11.4% (114/1,000) (≥40 IU/ml) and the GMC was 17.25 (95% CI: 15.49–19.03) IU/mL in the study population. There was statistical significance in the distribution of antibody levels in different age groups (*P* < 0.001), with the positive rate of anti-PT IgG antibody being 6.7% in the over-40 age group and 1.3% in the 20–29 age group (Table 1). The non-detectable rate (<5 IU/mL) of anti-PT IgG antibodies was 16.9, 17.7, 28.1, and 37.3% in 2018, 2019, 2020, and 2021, respectively. The non-detectable rate was higher during the post-COVID-19 pandemic (*p* < 0.05; Figure 1).

The GMC of each age group ranged between 14.15 (95% CI: 11.76–16.54) IU/ml and 23.52 (95% CI: 18.09–28.94) IU/ml, and there was no significant difference between the 30–39 age group and the ≥40 age group, but the GMC of the ≥40 age group was significantly higher than that of the 15–19 and 20–29 age groups after pairwise comparison (*p* < 0.001). A total of 29 (2.9%) subjects had anti-PT IgG concentrations ≥100 IU/ml, indicating the recent onset of infection, and 114 (11.4%) subjects had anti-PT-IgG antibodies concentrations ≥40 IU/ml, indicating a recent infection within a few years. Male and female difference in the positive rate and GMC were not statistically significant. In the Pre-COVID-19 era, the positive rate and GMC were much lower than Post-COVID-19 [5.4 vs. 19.1%, 14.68 (95% CI: 13.20–16.16) vs. 20.54 (95% CI: 16.98–24.09)], and the number of cases with post-COVID-19 anti-PT IgG antibody concentration ≥100 IU/ml increased significantly (Table 1).

The positive rate of anti-FHA IgG antibody in the study population was 20.2% (202/1,000) (≥40 IU/ml) and GMC was 24.94 (95% CI: 22.73–27.16) IU/mL, which were similar to anti-PT-IgG in age, sex, year, and COVID-19 pandemic (Table 2).

**Table 2:** The GMC and positivity rate of anti-FHA IgG antibodies in subjects aged ≥ 15 years.

	***N***	**GMC (95% CI) (IU/mL)**	***P-*value**	**Anti-FHA IgG (%)**			
				**≥40**	***P*-value**	**≥100**	***P-*value**
Age (years)			<0.001		<0.001		0.006
15–19	361	20.73 (18.07–23.38)		57 (15.8)		9 (2.5)	
20–29	223	24.25 (18.20–30.70)		44 (19.7)		6 (2.7)	
30–39	206	23.78 (19.25–28.31)		37 (18.0)		8 (3.9)	
≥40	210	33.18 (27.56–38.80)		64 (30.5)		17 (8.1)	
Sex			0.250		0.078		0.854
Male	411	23.39 (20.39–30.98)		72 (17.5)		17 (4.1)	
Female	589	26.03 (22.91–29.15)		130 (22.1)		23 (3.9)	
Year			<0.001		<0.001		<0.001
2018	212	17.28 (13.37–21.18)		22 (10.4)		2 (0.9)	
2019	348	20.97 (17.73–24.21)		53 (15.2)		4 (1.1)	
2020	217	40.51 (35.30–45.73)		79 (36.4)		19 (8.8)	
2021	223	23.40 (18.05–28.74)		48 (21.5)		15 (6.7)	
COVID-19			<0.001		<0.001		<0.001
Pre-COVID-19	560	19.57 (17.07–22.06)		75 (13.4)		6 (1.1)	
Post-COVID-19	440	31.84 (28.03–35.65)		127 (28.9)		34 (7.7)	
Total	1,000	24.94 (22.73–27.16)		202 (20.2)		40 (4.0)	

CI, confidence interval.

### Assessment of actual infection

We assumed that the infection occurred within 58.6 days, and based on the overall proportion (2.9%) of individuals with anti-PT IgG antibody ≥100 IU/ml, the incidence rate (/100) was estimated by the formula to be 18.08 (95% CI: 12.40–26.11). There is about 70 million residents who were ≥15 in Jiangsu Province, and the estimated number of cases was close to 12 million, but only 15 cases (≥15 years, Unpublished data) were reported in these 4 years. The estimated incidence rates (/100) of pertussis among people aged ≥15 years in 2018 were 5.36 (95% CI: 1.37–16.89). In 2020, that number was as high as 36.34 (95% CI: 20.38–62.27). Furthermore, the estimated incidence of Post-COVID-19 was higher than that of Pre-COVID-19 (36.33/100 vs. 12.84/100), and the difference was statistically significant (*p* < 0.05; Table 3).

**Table 3:** Estimated incidence of pertussis in healthy people ≥15 years of age.

	**Reported cases (*N*)**	**Anti-PT IgG (+)**	**Sero-prevalence (%, 95% CI)**	Estimated incidence/100 (95% CI)^*^
**Year**
2017	57	2	0.94 (0.16–3.73)	5.86 (0.99–23.21)
2018	137	3	0.86 (0.22–2.71)	5.36 (1.37–16.89)
2019	258	11	5.07 (2.69–9.13)	31.60 (16.77–56.91)
2020	14	13	5.83 (3.27–9.99)	36.34 (20.38–62.27)
**COVID-19**
Pre-COVID-19	452	16	2.06 (1.27–3.32)	12.84 (7.91–20.72)
Post-COVID-19	14	13	5.83 (3.44–9.72)	36.33 (21.44–60.58)
Total	466	29	2.9 (1.99–4.19)	18.08 (12.40–26.11)

CI, confidence interval.

* The rate of pertussis infection can be estimated using the formula: 365.25/58.6 × Sero-prevalence ≥100 IU/ml, but it represents the rate 1 year before, the numbers for 2021 are used to estimate the incidence for 2020, and so forth. Data for 2022 were unknown and we were unable to estimate the incidence for 2021, so we included only 2020 values in estimating the incidence Post-COVID-19.

## Discussion

In the era before COVID-19 pandemic, pertussis was one of the primary global public health issues worldwide. Vaccination coverage was more than 85% for children born after 1990, while vaccination coverage was low for children born before 1990, and there was no introduction of booster dose for adolescents and adults in China (22–24). It is well-known that the duration of immunity in children after vaccination is estimated to wane over 4–12 years (25). Seroepidemiological data on pertussis observed nationally and internationally suggested that vaccine-acquired immunity had waned in adolescents and adults, and the majority of subjects enrolled in this study had been vaccinated for more than 15 years. Therefore, subjects with antibodies ≥40 IU/mL were considered to have pertussis infection within a few years. However, pertussis in adolescents and adults often presents with atypical symptoms such as asymptomatic infection and chronic cough (15–17). Moreover, the fact that most cases of pertussis in China were based on clinical rather than laboratory diagnosis, so the prevalence of pertussis in China may be grossly underestimated.

The present study found that antibody concentrations of anti-PT IgG and anti-FHA IgG increased with age, but the current findings cannot be attributed to childhood vaccination, implying an underlying Bordetella pertussis infection. Additionally, the results of this study also showed that the positive rate and GMC of anti-FHA IgG were both generally higher than those of anti-PT IgG. One possible explanation for this pattern was that the antibody response to FHA was not specific to Bordetella pertussis infection. Unlike anti-PT IgG, which was specifically produced after contact with Bordetella pertussis, other infections such as respiratory pathogens, Bordetella parapertussis and Haemophilus influenzae caused cross-antigen reactions that also inducing the production of anti-FHA IgG (26–29), and the kinetic studies had revealed a slower decline of anti-FHA IgG than anti-PT IgG (30).

Since China's pertussis vaccination policy has not changed since the 1980s, infants were primarily administered with three doses of DTwP vaccines, at age of 3, 4, 5 months, and a booster dose is given at 18–24 months. The adults of childbearing age with anti-PT IgG antibodies ≥40 IU/mL were considered to have a real B. pertussis infection. The number of pertussis cases increased significantly in the Pre-COVID-19 era, and the reported age of pertussis had shifted from infants and young children to all age groups, with a notable rise in cases among adults of reproductive age. Following the outbreak of COVID-19 pandemic, the implementation of a series of non-pharmacological interventions (lockdown, wearing masks, maintaining social distance, etc.) had a significant effect on diseases caused by respiratory infections, and only 14 pertussis cases were reported in Jiangsu Province in 2020. Interestingly, our seroprevalence results showed that the GMC and positivity rate of anti-PT IgG antibodies in subjects were significantly higher in post-COVID-19 than in pre-COVID-19 [19.1% vs 5.4%, 20.54 (95% CI: 16.98–24.09) vs. 14.68 (95% CI: 13.20–16.16)], and the anti-PT IgG antibody concentration ≥100 IU/ml significantly increased post-COVID-19. The Chinese government has been following a policy of dynamic reset for epidemic prevention, and in order to achieve this goal, many cities will choose static management when new coronavirus transmission, while also requiring residents to regularly collect nucleic acid samples at predetermined locations. Large groups will assemble during sample, people will chat while waiting for sampling, and some respiratory infections could spread since people won't properly follow epidemic prevention measures like keeping a social distance and wearing masks. The majority of adults in China do not have a history of vaccination against pertussis, thus returning home after sampling can spread the disease among family members.

There were about 70 million residents aged ≥ 15 years in Jiangsu Province, and the estimated number of cases was close to 12 million, but only 15 cases (>15 y) were reported from 2018 to 2021, indicating that the actual incidence of pertussis in Jiangsu province was much higher than officially reported. The actual incidence in Israel was 400 higher than the number of officially reported cases; the figures for the Netherlands and Estonia were 600 and 900 times higher, respectively (17, 21, 31). The estimated population prevalence of pertussis in Chongqing was 7,290/100,000 (32), and that in Guangdong province was 9,395/100,000 for people aged ≥7 years (33). It is well-known that vaccination is the most effective tool for the prevention of pertussis. Considering the changing epidemiological pattern of pertussis (1), there was a need for booster dose vaccination of adults and adolescents including maternal immunization. However, no booster immunizations have been recommended in China to date.

There were some limitations to this study. First, the subjects' vaccination history was unclear. Secondly, Most of the people who attended health examinations were healthy and did not have cough or other symptoms. Thirdly, the sensitivity of PCR varies in different stages of the disease, which affects the results of our study to some extent.

## Conclusions

Our study showed that the number of reported cases of pertussis declined sharply after the COVID-19 pandemic, but the sero-prevalence of pertussis in the population was higher than in the pre-COVID-19 pandemic era, and these results confirming the high rate of under-reporting of pertussis in the Jiangsu. In addition, the proportion of adults without pertussis-specific antibodies has remained high since the COVID-19 pandemic, suggesting that a large number of adults of childbearing age are susceptible to pertussis. Furthermore, given the high estimated incidence of pertussis among adolescents and adults, it seems imperative that vaccination of adolescents and adults should be considered for inclusion in vaccination programs.

## Data availability statement

The raw data supporting the conclusions of this article will be made available by the authors, without undue reservation.

## Ethics statement

The studies involving human participants were reviewed and approved by the Medical Ethics Committee of Jiangsu of the Jiangsu Provincial Center for Disease Control and Prevention. Written informed consent to participate in this study was provided by the participants' legal guardian/next of kin.

## Author contributions

XSu and HS conceived of this study. QC, XSh, and WW collected the data. YZ, YW, and YX analyzed the data and drafted initial manuscript. XSu, HS, WW, and ZW critically revised the manuscript and helped interpret data. All authors reviewed the final manuscript.

## Funding

This work was supported by the Preventive Medicine Association of Jiangsu Provincial Health and Family Planning Commission (Grant No. Y2018072).

## Conflict of interest

The authors declare that the research was conducted in the absence of any commercial or financial relationships that could be construed as a potential conflict of interest.

## Publisher's note

All claims expressed in this article are solely those of the authors and do not necessarily represent those of their affiliated organizations, or those of the publisher, the editors and the reviewers. Any product that may be evaluated in this article, or claim that may be made by its manufacturer, is not guaranteed or endorsed by the publisher.
